# Concurrent intra-arterial carboplatin administration and radiation therapy for the treatment of advanced head and neck squamous cell carcinoma: short term results

**DOI:** 10.1186/1471-2407-9-313

**Published:** 2009-09-04

**Authors:** Giulia Bertino, Antonio Occhini, Carlo Emilio Falco, Camillo Porta, Franco Corbella, Sara Colombo, Vittoria Balcet, Patrizia Morbini, Federico Zappoli, Andrea Azzaretti, Giuseppe Rodolico, Carmine Tinelli, Marco Benazzo

**Affiliations:** 1 Department of Otolaryngology Head Neck Surgery, University of Pavia, IRCCS Policlinico S. Matteo Foundation, P.le Golgi 2, 27100 Pavia, Italy; 2 Institute of Clinical Oncology, University of Pavia, IRCCS Policlinico S. Matteo Foundation, P.le Golgi 2, 27100 Pavia, Italy; 3 Institute of Radiation Oncology, IRCCS Policlinico S. Matteo Foundation, P.le Golgi 2, 27100 Pavia, Italy; 4 Institute of Pathology, University of Pavia, IRCCS Policlinico S. Matteo Foundation, P.le Golgi 2, 27100 Pavia, Italy; 5 Radiodiagnostic Unit, IRCCS Policlinico S. Matteo Foundation, P.le Golgi 2, 27100 Pavia, Italy; 6 Clinical Epidemiology and Biometric Unit, IRCCS Policlinico S. Matteo Foundation, P.le Golgi 2, 27100 Pavia, Italy

## Abstract

**Background:**

The aim of the present study was to evaluate the survival, efficacy and safety of a modified RADPLAT-like protocol using carboplatin instead of cisplatin.

**Methods:**

Fifty-six patients with primary head and neck squamous cell carcinoma received 4 cycles of intra-arterial carboplatin (350 mg/m^2 ^per cycle every 2 weeks), with concurrent three-dimensional conformal radiation therapy.

**Results:**

Two major and 4 minor complications were observed. Forty-five of the 56 patients (80%) completed the protocol, while 11 (20%) patients had to discontinue the intra-arterial infusions due to the occurrence of severe haematological toxicity, but were able to complete radiotherapy.

Forty-four (98%) of the 45 patients who completed the protocol and 10 (91%) of the 11 who did not, were free of disease at the end of the treatment, for a comprehensive 96% of CR overall.

After a median 23.55 months (range: 2 to 58 months) of follow-up, 40 patients (71%) are alive and disease-free, 1 (2%) is alive but affected by disease and 15 (27%) have died of the disease or other causes.

**Conclusion:**

Intra-arterial carboplatin administration with concurrent three-dimensional conformal radiation therapy seems to be a promising alternative to RADPLAT in the treatment of advanced head and neck tumours. Haematological and non-haematological toxicities are virtually similar, but carboplatin has the advantage in that it is not nephrotoxic and can be used at very high doses without any significant increase in the extent of side effects.

## Background

During the past decade, chemoradiation has become the most important treatment option for locally advanced head and neck squamous cell carcinoma (HNSCC). In particular, the combination of concurrent chemotherapy and three-dimensional conformal radiotherapy (3D CRT) appears to be more potent than sequential chemoradiation therapy in terms of local control of the disease and improvement of overall survival [1,2]. To overcome the potential chemoradioresistance of head head and neck tumours, supradose infusions of cisplatin via superselective intra-arterial chemotherapy were introduced [3-5]. Thanks to the convincing results of Robbins' RADPLAT protocol [5], many other institutions have used RADPLAT or RADPLAT mimicking protocols for the treatment of HNSCC, with a high success rate [6-8].

To overcome the main cisplatin-related toxicities, i.e. kidney function impairment and nausea/vomiting [9], many authors have replaced cisplatin with carboplatin, which has proven to be as effective as cisplatin in sequential or concurrent chemoradiation protocols, but better tolerated; indeed, carboplatin is virtually non-nephrotoxic and does not cause such severe nausea and vomiting [10-12].

For this reason, we first applied Robbins' super-selective intra-arterial administration technique to infuse carboplatin in a neo-adjuvant treatment protocol with very encouraging results [13-15].

The low rate of toxic effects observed in our first protocol [15] led us to initiate a phase II protocol combining 3D CRT with concomitant intra-arterial carboplatin administration to treat advanced HNSCC.

The aim of this study was to evaluate survival, efficacy and safety of our modified RADPLAT-like protocol on a single group of patients affected by advanced HNSCCC.

## Methods

### Patient characteristics

Fifty-six patients (46 men and 10 women), aged from 38 to 74 years (mean 58,4, median 59 years), with previously untreated squamous cell carcinoma (SCC) of the upper aerodigestive tract were treated between March 2003 and February 2008. The sites and stages of the tumor, classified according to the UICC/AICC TNM [16], are reported in table 1 and table 2. The stage II patients in table 2 were submitted to this protocol owing to general conditions that contraindicated surgery or because they refused to undergo surgery. Patients were required to sign an informed consent form after the Ethical Committee of our Institution formally approved the study.

**Table 1:** Site of the tumor

Tumor site	N.
Oral cavity	20

Hypopharynx	3

Oropharynx	32

Larynx	1

**Total**	56

**Table 2:** Stage of the tumor

Stage	TNM	N.
II	T2N0M0	12

III	T2N1M0	6
	T3N0M0	9
	T3N1M0	4

IVA	T1N2aM0	1
	T1N2bM0	3
	T2N2aM0	4
	T2N2bM0	1
	T3N2aM0	1
	T3N2bM0	1
	T3N2cM0	2
	T4N0M0	3
	T4N1M0	2
	T4N2aM0	3
	T4N2bM0	1
	T4N2cM0	2

IVB	T2N3M0	1

**Total**		56

### Pre-treatment evaluation

Patients underwent a complete clinical and laboratory examination including measurement of haematological, hepatic and renal parameters, as well as general metabolic functions, chest radiography and electrocardiography. The disease was staged by physical and endoscopic examination, biopsy with histopathological and immunohistochemical examination, CT scan of the head and neck and of the thorax, MRI (when appropriate) and PET.

Inclusion criteria for the treatment protocol were primary untreated SCC, absence of distant metastases, age < 74 years, no haematological or metabolic contraindications against chemotherapy.

### Treatment protocol

**Figure 1:**
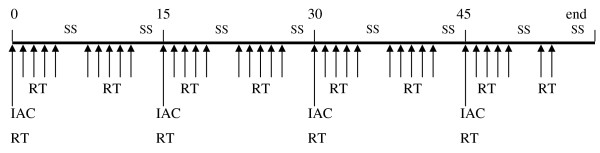
**Timing of the treatment protocol**. SS = Saturday, Sunday; RT = radiotherapy; IAC = intra-arterial chemotherapy.

Figure 1 summarizes the timing of the treatment protocol. The planned dose of carboplatin per cycle was 350 mg/m^2 ^with a maximum amount of 1,4 g/m^2 ^given in 4 cycles, every 2 weeks. No pharmacokynetic analyses were performed to select the dose of the drug, but it was chosen according to our previous experience of induction intra-arterial chemotherapy [15]. Carboplatin was administered intra-arterially (i.a.), dissolved in 80 mL of 0,9% saline and infused over 15-20 min by a battery-operated pump (Medrad Mark 5 Plus) with an infusion velocity of 4-6 mL/min. An angiographic catheter (Glidecath Radicofocus 5F Terumo) was introduced percutaneously under local anesthesia (10 mL of 2% lidocaine solution) into the common femoral artery, according to the Seldinger technique. The catheter tip was placed in the external carotid artery, under radiographic control. Diagnostic transfemoral carotid arteriography was performed and the most suitable branch of the artery, providing the main blood supply to the tumour, was selectively catheterised. The catheter was removed immediately after the infusion. A simultaneous intravenous (i.v.) infusion of 250 mL of 0,9% saline was administered. Dexamethasone (4 mg) and ondansetron (1 g) dissolved in 100 mL of 0,9% saline were administered i.v. 1 hour before the infusion.

While undergoing chemotherapy, patients also received concomitant 3D CRT with conventional fractionation (1,8 - 2 Gy per fraction, five days per week). The total dose was 66 - 74 Gy for PTVI (Planning Target Volume: volume planned to receive prescribed dose, according to ICRU Report 50 and 62) [17,18] including tumour site and clinically positive nodes, and 50-60 Gy for PTVII, including tumour site and all cervical lymph nodes (positives and negatives).

When the volume including tumor and positive nodes resulted too large to receive 64-72 Gy (9 cases), we defined three PTV: PTV I including tumor site, PTV II including tumour site and positive nodes, PTV III including tumor site and all cervical lymph nodes (positives and negatives). In these cases we had 64-74 Gy for PTV I, 56-66 Gy for PTV II, 50-60 Gy for PTV III.

All patients underwent planning CT in a supine position with a thermoplastic mask as a custom immobilisation device. In all slices acquired planning target volumes and organs at risk (spinal cord, parotid glands, mandibula) were contoured. Dose-volume histograms (DVH) were evaluated by the radiation oncologist and physicians and only dose inhomogenities between 90% and 110% out of prescribed dose were accepted. All patients were irradiated on a linear accelerator, with Cerrobend blocks or multileaf collimators. All treatment fields were verified before starting therapy.

### Treatment evaluation

Haematological and non-haematological toxicities were evaluated according to NCI-CTCAE v3.0 [19] 24 hours before and after each course of i.a. chemotherapy. Response to therapy was assessed at the end of the treatment protocol by physical examination and endoscopy, while CT scan and/or MRI were performed at 3, 6, and every 12 months after the end of therapy; a biopsy was performed if recurrence was suspected. A complete response (CR) was defined as the complete disappearance of all demonstrable lesions. A partial response (PR) was defined as a decrease of 50% or more in the sum of the products of the largest perpendicular diameters of all measurable lesions. No response (NR) was defined as a regression of < 50% in total tumour size or as stable or progressive disease.

### Statistical analysis

The first end point was overall survival. Survival was measured from the date of the first infusion to the date of death or the date when the patient was last known to be alive. Secondary end points were CR rates, loco-regional tumour control and disease-free survival. Time to loco-regional failure was measured from the start of treatment and the date of disease relapse, the date of disease-related death, or the date when the patient was last known to be alive and disease-free. Loco-regional failure, distant metastases, or disease-related death were all considered failures for disease-free survival. OS and disease-free survival rates (with their 95% Confidence Intervals) were estimated with the Kaplan-Meier method and log rank tests were used for univariate comparisons between groups. P values lower than 0.05 were considered significant. Data analysis was performed with the STATA statistical package (release 9.0, 2006, Stata Corporation, College Station, Texas, USA).

## Results

### Feasibility

Two hundred and thirteen super-selective transfemoral intra-arterial infusions of carboplatin were performed. Two major complications (1 transient ischemic attack (TIA) and 1 partial necrosis of the tongue) and 4 minor complications (3 cases of skin rash and 1 neck oedema) linked to the intra-arterial chemotherapy were observed.

### Toxicity

Carboplatin treatment was well-tolerated by the majority of the patients. In particular, 45 of the 56 patients (80%) completed the protocol, while 11 (20%) patients had to discontinue the intra-arterial infusions, but nevertheless were able to complete radiotherapy.

Table 3 summarizes why the infusions were suspended. In 1 case, the patient decided to interrupt intra-arterial chemotherapy while in another case it was suspended due to the occurrence of bronchopneumonia. In the other 9 cases, chemotherapy was suspended due to the appearance of thrombocytopenia. This was associated with leukopenia and anaemia in 5 of the 9 patients.

**Table 3:** Reasons for suspension of intra-arterial chemotherapy.

N. of infusions	N. of patients	Reasons for suspension		
1/4	1	pneumonia		

2/4	1	thrombocytopenia (1)	leukopenia (4)	anaemia (3)

	1	thrombocytopenia (4)	leukopenia (1)	anaemia (1)

3/4	1	Patient's decision		

	2	thrombocytopenia (1)	leukopenia (1)	

		thrombocytopenia (1)	leukopenia (1)	anaemia (1)

	2	thrombocytopenia (2)	leukopenia (1)	

		thrombocytopenia (2)	leukopenia (1)	anaemia (1)

	2	thrombocytopenia (3)	leukopenia (3)	

		thrombocytopenia (3)	leukopenia (2)	anaemia (3)

	1	thrombocytopenia (4)		

**Total**	11			

(n.) = grade of haematological toxicity (NCI-CTCAE v3.0)

Tables 4 and 5 list all cases of haematological and non-haematological toxicity observed during the treatment protocol.

**Table 4:** Acute haematological toxicity (NCI-CTCAE v3.0)

	Grade 1 (n.v. - 10 g/dl)	Grade 2 (<10 - 8 g/dl)	Grade 3 (< 8 g/dl - 6,5 g/dl)	Grade 4 (< 6,5 g/dl)
Anaemia*	20/56 (35%)	6/56 (11%)	3/56 (5%)	0/56 -

	**Grade 1** **(n.v. - 1,5 × 10^3^/μl)**	**Grade 2** **(<1,5 - 1 × 10^3^/μl)**	**Grade 3** **(< 1 - 0,5 × 10^3^/μl)**	**Grade 4** **(< 0,5 × 10^3^/μl)**

Leukopenia*	14/56 (25%)	10/56 (18%)	8/56 (14%)	1/56 (2%)

	**Grade 1** **(n.v. - 75 × 10^3^/μl)**	**Grade 2** **(< 75 - 50 × 10^3^/μl)**	**Grade 3** **(< 50 - 25 × 10^3^/μl)**	**Grade 4** **(< 25 × 10^3^/μl)**

Thrombocytopenia*	10/56 (18%)	6/56 (11%)	5/56 (9%)	2/56 (3%)

*worst toxicity observed

**Table 5:** Non-haematological acute toxicity (NCI-CTCAE v3.0) *worst toxicity observed

	Grade 1 (erithema)	Grade 2 (ulcers or pseudomembranes)	Grade 3 (confluent ulcers)	Grade 4 (necrosis)
Stomatitis*	15/56 (27%)	12/56 (21%)	4/56 (7%)	1/56 (2%)

	**Grade 1** (symptomatic, regular food intake)	**Grade 2** (impaired swallowing; i.v. fluids)	**Grade 3** (enteral/parenteral nutrition)	**Grade 4** (life-treatening consequences)

Dysphagia*	20/56 (36%)	10/56 (18%)	4/56 (7%)	0/56 -

	**Grade 1** (light erithema or desquamation)	**Grade 2** (moderate erithema or desquamation with oedema)	**Grade 3** (erithema or desquamation with induced bleeding)	**Grade 4** (necrosis with spontaneous bleeding)

Dermatitis*	14/56 (25%)	3/56 (5%)	3/56 (5%)	0/56 -

	**Grade 1** (asymptomatic)	**Grade 2** (symptomatic without respiratory distress)	**Grade 3** (stridor, respiratory distress)	**Grade 4** (severe compromission, tracheotomy)

Laryngeal oedema*	10/56 (18%)	0/56 -	0/56 -	2/56 (3%)

	**Grade 1** (light)	**Grade 2** (moderate)	**Grade 3** (severe)	**Grade 4** (disabling)

Pain*	10/56 (18%)	5/56 (9%)	0/56 -	0/56 -

	**Grade 1** (flushing, or transient rash, fever < 38°)	**Grade 2** (flushing, rash or orticaria, fever = 38°)	**Grade 3** (bronchospasm with or without orticaria)	**Grade 4** (anaphylaxis)

Allergic reactions*	1/56 (2%)	2/56 (3%)	0/56 -	0/56 -

	**Grade 1** (loss of appetite)	**Grade 2** (lower caloric intake without weight loss)	**Grade 3** (weight loss requiring enteral/parenteral nutrition)	**Grade 4** (life-threatening denutrition)

Nausea*	13/56 (23%)	0/56 -	0/56 -	0/56 -

	**Grade 1** (no treatment required)	**Grade 2** (necessity of brief liquid i.v infusion)	**Grade 3** (necessity of continuative treatment > 24 h)	**Grade 4** (shock)

Hypotension*	1/56 (2%)	2/56 (3%)	0/56 -	0/56 -

	**Grade 1**	**Grade 2**		

Alopecia*	2/56 (3%)	0/56 -		

Two patients underwent s.c. granulocyte-colony stimulating factor (G-CSF) administration due to the appearance of severe leukopenia and 1 patient received erithrocyte transfusion for severe anaemia all after 3 infusions; 2 patients required tracheotomy due to severe dyspnea; 1 patient was temporarily fed with a nasogastric tube; while 3 other patients were submitted to PEG for severe mucositis with odynophagia. All the non-haematological complications appeared after 20 days from the beginning of the treatment protocol

### Response rate

**Figure 2:**
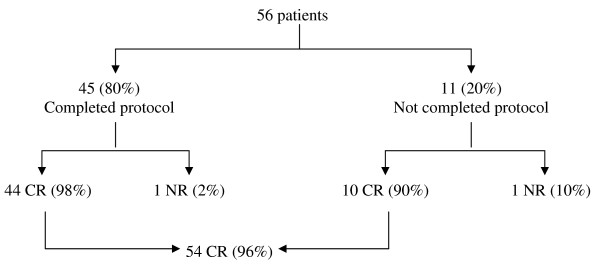
**Immediate post-treatment response**. CR = complete response; NR = no response.

Figure 2 summarizes the immediate post-treatment response. Forty-four (98%) of the 45 patients who completed the protocol and 10 (91%) of the 11 who did not, were free of disease at the end of the treatment, for a comprehensive 96% of CR.

### Disease control and survival

No patients were lost to follow-up. Median follow-up time (25^th^-75^th ^percentile), measured from the first intra-arterial carboplatin administration, was 23.55 months (range: 2 to 58 months).

The 2 NR patients died of the disease after 9 and 21 months from the beginning of the treatment; of the other 54 CR patients, 1 died of broncopneumonia 9 months after the treatment beginning; 14 recurred after a median period of 9 months (range 4-19 months). In 5 of these 14 patients disease recurred at the primary site, 2 at the primary site and on the neck, 1 at the primary site with pulmonary and bone metasases, 1 on the neck with pulmonary metasases, 4 presented only distant metasases; 1 patient developed a second primary tumour. All the patients who presented regional recurrence and/or distant metastases had clinically positive necks at the beginning of therapy.

**Figure 3:**
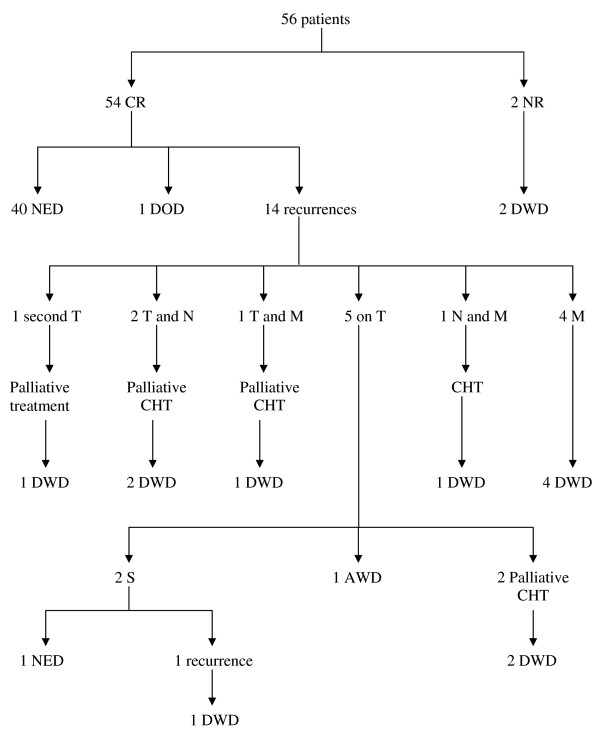
**Follow-up**. CR = complete response; NR = no response; NED = no evidence of disease; DOD = died of other disease; DWD= died with disease; T = tumour; N = neck; M = Metastasis; CHT = chemotherapy; S = surgery; AWD = alive with disease.

Only 2 of the 5 patients with recurrence at the primary site were submitted to salvage surgery, both 10 months after the beginning of the treatment; 1 of them is still alive and disease free but the other developed disease recurrence 3 years later and died of the disease. One patient with recurrence at the primary site is still alive with disease. The other 9 patients with recurrence were all submitted to systemic chemotherapy (curative or palliative) and died of disease (Figure 3).

In conclusion, 40 patients (71%) are alive and disease-free (NED), 1 (2%) is alive with disease (AWD) and 15 (27%) have died of the disease (DWD) or other causes (DOD).

The percentage of organ preservation was 97,5%, as only 1 of the 40 NED patients underwent salvage surgery at the tumour site.

**Figure 4:**
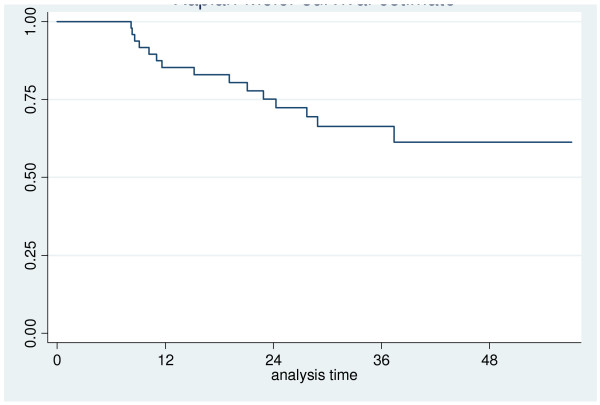
**Overall survival**.

**Figure 5:**
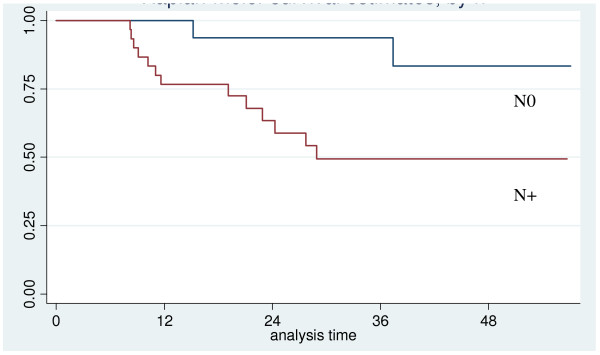
**Overall survival according to neck status**.

**Figure 6:**
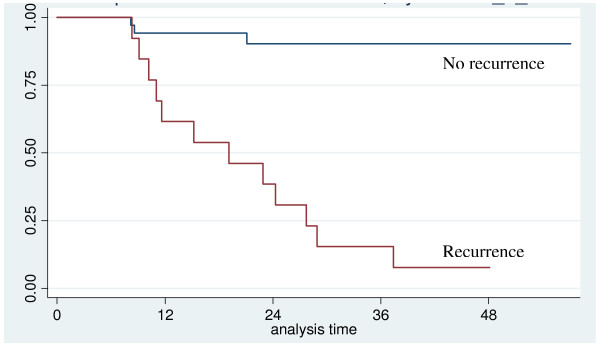
**Overall survival according to recurrence**.

The probability of two-year overall survival was 75% (95% CI: 59% - 85%) (Figure 4). Among the factors influencing survival, gender, N status and recurrence were statistically significant. In particular, all the patients who died were male and the presence of a positive neck at the time of diagnosis and/or recurrence were significantly negatively correlated with survival (Figure 5 and 6).

Age, tumour site, T-stage, grading and completion of the treatment showed no effect on survival.

**Figure 7:**
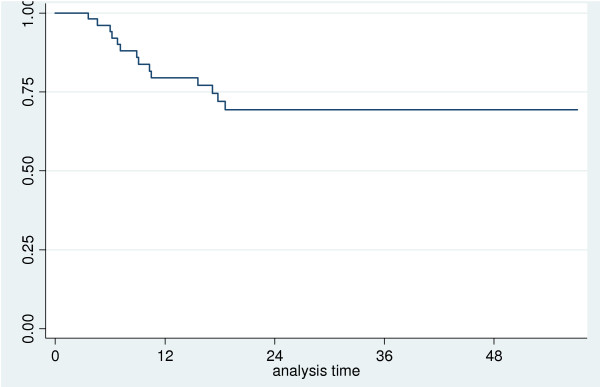
**Disease free survival**.

Two-year disease-free survival was 69% (95% CI: 54% - 81%) (Figure 7). In this case, the only factor significantly influencing the appearance of recurrence was gender, in fact all the patients who recurred were males.

As regards T and N stage, we observed a higher likelihood of recurrence for advanced tumours, even if this tendency was not statistically significant. Other factors such as age, tumour site, grading and completion of the treatment did not significantly influence recurrence of the disease.

## Discussion

Our study confirmed the evidence that intra-arterial chemotherapy by superselective transfemoral infusion of high doses of platinum compounds with concomitant 3D CRT is feasible and can be easily reproduced, with minimal procedural complications [6].

We chose carboplatin and its dosage in view of the efficacy of its anti-tumoral activity demonstrated in our previous reports [13-15]. The dose of 350 mg/m^2 ^was comparable to the cisplatin "decadoses" used by Robbins et al. [3,9,20-22], without relying on supportive measures to alleviate cisplatinum-related toxicities.

Compared with our previous neo-adjuvant intra-arterial carboplatin protocol [15], systemic toxicity of concurrent intra-arterial carboplatin administration and 3D CRT was usually mild to moderate; in fact, 11 (20%) patients did not complete the four cycles of chemotherapy due to the appearance of haematological toxicity, involving all three blood-cell lines, platelets, leukocytes and red blood cells. In particular, severe thrombocytopenia was the factor which mostly limited intra-arterial infusion owing to the high risk of haemorrage during the Seldinger procedure. Local toxicity also occurred frequently, mainly affecting the skin and oral mucosa, requiring tracheotomy in 2 patients, nasogastric tube feeding in 1 patient and PEG in another 3 patients.

These results are similar to those presented by other authors who used significantly lower doses of carboplatin [10,11] and suggest that carboplatin dose-increase does not worsen toxic effects. For this reason, we believe that carboplatin could be used as an alternative to cisplatin in RADPLAT mimicking protocols [12], instead of performing a less intensive RADPLAT treatment, as proposed by Yoshizaki et al. [8].

Even though the results of all the concurrent chemoradiotherapy trials are difficult to compare due to differences regarding dose, timing of intra-arterial infusion and duration of follow-up [5-7,23,24], our study confirmed that the percentage of post-treatment complete response is very high (96%) (table 6) and superior to induction intra-arterial chemotherapy protocols [15,25,26].

**Table 6:** Literature review

Author	Protocol	Response	Toxicity	Overall survival	Disease free survival
Robbins KT, et al. (2000)	Cisplatin 150 mg/m^2 ^week × 4 + RT 68-72 Gy	CR 80% T CR 61 N	89 pts. grade III-IV; 6 grade V	38.8% 5-yrs	53.6% 5 yrs

Regine WF, et al. (2001)	Cisplatin 150 mg/m^2 ^week × 2 + RT 78-82 Gy	CR 88% T CR 85% N	69% grade III 7% grade IV	57% 2 yrs	63% 2 yrs

Robbins KT, et al. (2005)	Cisplatin 150 mg/m^2 ^week × 4 + RT 70 Gy	CR 85% T CR 88% N	44% grade III 39% grade IV 3% grade V	63% 2 yrs	46% 2 yrs

Rabbani A, et al. (2007)	Cisplatin 150 mg/m^2 ^week × 4 + RT 70 Gy		72% grade III, IV; 6% grade V	57% 4 yrs	65% 4 yrs

Present study	Carboplatin 350 mg/m^2 ^2 weeks × 4 + RT 66-74 Gy PTVI - 50-60 Gy PTVII	CR 96%	30% grade III, IV	75% 2 yrs	69% 2 yrs

However, part of the impressive effect of this kind of protocol could be due also to the fact that 12 of the 56 patients (21%) were in limited stage II.

This therapeutic regimen also has impressive organ preservation capabilities [1,12] as confirmed by 97.5% of our NED patients in whom the tumour site was preserved.

Our two-year overall survival rate of 75% was comparable to that observed in other RADPLAT protocols with the same follow-up period [8,12]. As pointed out by other authors [1,27-29], N stage and the recurrence of the disease were the most important factors influencing overall survival. In fact, all our patients in whom neck disease recurred or who developed distant metastases had clinically positive necks at the time of diagnosis. For this reason, many authors suggest planning neck dissection in N+ patients submitted to concurrent intra-arterial chemoradiotherapy [1,30-32].

Surprisingly, we found that another factor conditioning survival was gender. We are unable to explain this evidence; genetic factors may influence the biological response of the tumour to treatment, or male patients may keep up habits that can influence recurrence of the disease, such as smoking and alcohol consumption.

The Kaplan-Meyer estimate of two-year disease free survival was 69%. Recurrence and death due to the disease occurred within the first 24 months after treatment; for this reason we recommend a more thorough follow-up during this period so that appropriate treatment can be implemented as soon as possible, even though the results of salvage surgery and/or systemic chemotherapy are often frustrating.

Also in the case of disease-free survival, all the patients in whom disease recurred were males. The explanation may be the same as for overall survival. Unfortunately, there are no data in the literature that help to explain this phenomenon.

Regarding the role of haematological stimulating factors in conditioning survival, even if randomized trials have demonstrated that they can have a negative impact on prognosis in patients with HNSCC submitted to chemo-radiotherapy [33,34], our study didn't give definitive results because only 2 patients were treated with these compounds and 1 is disease-free and the other died for disease. For this reason, the use of growth factors during radiochemotherapy is questionable and their possible negative effect must be taken into consideration when analizing survival of this kind of treatment protocols.

## Conclusion

Intra-arterial carboplatin administration with concurrent three-dimensional conformal radiation therapy seems to be an effective treatment of advanced head neck tumours at least in a short time of follow-up. Moreover, the haematological and non-haematological toxicities virtually similar to the RADPLAT protocol, render carboplatin a valid alternative because it has the advantage of not being nephrotoxic and being used at very high doses without any significant increase in the entity of side effects.

## Competing interests

The authors declare that they have no competing interests.

## Authors' contributions

GB prepared and edited the manuscript. AO, CP, FC, FZ, were involved in revising results and in preparing the different topics of the manuscript (surgery, radiotherapy, chemotherapy and infusional technique). CEF prepared the data base and collected all the data. CT performed the statistical analysis. SC and VB performed radiation protocols for the patients; PM performed all the histopathological and immunohistochemical analyses; AA and GR performed all the intra-arterial infusions. MB, chief of the department, revised and gave final approval of the version of the manuscript. All authors read and approved the final version of the manuscript.

## Pre-publication history

The pre-publication history for this paper can be accessed here:

http://www.biomedcentral.com/1471-2407/9/313/prepub
